# Genetic polymorphisms of *GGT1* gene (rs8135987, rs5751901 and rs2017869) are associated with neoadjuvant chemotherapy efficacy and toxicities in breast cancer patients

**DOI:** 10.1186/s12920-023-01685-7

**Published:** 2023-10-27

**Authors:** Lu Sun, Ziping Wu, Yanping Lin, Shuguang Xu, Yumei Ye, Wenjin Yin, Liheng Zhou, Jingsong Lu

**Affiliations:** 1 Department of Breast Surgery, School of Medicine, Renji Hospital, Shanghai Jiao Tong University, NO.160 Pujian Road, Shanghai, 200127 China; 2 Present Address: Department of Gynaecologic Oncology, Zhejiang Cancer Hospital, Hangzhou Institute of Medicine (HIM), Chinese Academy of Sciences, Hangzhou, 310022 Zhejiang China

**Keywords:** *GGT1* gene, Single nucleotide polymorphism, Breast cancer, Neoadjuvant therapy

## Abstract

**Background:**

Our previous study illustrated the predictive value of serum gamma-glutamyl transpeptidase (GGT) for neoadjuvant chemotherapy (NAC) sensitivity in breast cancer patients. In this study we aim to determine whether single nucleotide polymorphisms (SNPs) in the gamma-glutamyltransferase 1 (*GGT1*) gene are related to the NAC response and adverse events and to find out a genetic marker in predicting NAC sensitivity.

**Methods:**

Three SNP loci (rs8135987, rs5751901, rs2017869) of *GGT1* gene were selected and tested among breast cancer patients reciving NAC. Four genotype models were used in SNP analysis: co-dominant model compared AA vs. Aa vs. aa; dominant model compared AA vs. Aa + aa; recessive model compared AA + Aa vs. aa; over-dominant model compared AA + aa vs. Aa. Chi-squared test and multivariable logistic regression analysis were performed between SNP genotypes, haplotypes and pathological complete response(pCR), adverse events as well as serum GGT level.

**Results:**

A total of 143 patients were included in the study. For SNP rs8135987 (T > C), the TC genotype in over-dominant model was inversely related with pCR (adjusted OR = 0.30, 95% CI 0.10–0.88, *p* = 0.029) as well as the risk of peripheral neuropathy (adjusted OR = 0.39, 95% CI 0.15–0.96, *p* = 0.042). The TC genotype in dominant model was significantly associated with elevated serum GGT level (OR = 3.11, 95% CI 1.07–9.02, *p* = 0.036). For rs2017869 (G > C), the occurrence of grade 2 or greater neutropenia (OR = 0.39, 95% CI 0.08–0.84, *p* = 0.025) and leukopenia (OR = 0.24, 95% CI 0.08–0.78, *p* = 0.017) were both significantly reduced in patients with CC genotypes. For rs5751901(T > C), the CC genotype could significantly reduce the risk of grade 2 or greater neutropenia (OR = 0.29, 95% CI 0.09–0.96, *p* = 0.036) and leukopenia (OR = 0.27, 95% CI 0.09–0.84, *p* = 0.024) in recessive model.

**Conclusions:**

The *GGT1* gene SNPs might be an independent risk factor for poor response of NAC in breast cancer patients, providng theoretical basis for further precision therapy.

**Supplementary Information:**

The online version contains supplementary material available at 10.1186/s12920-023-01685-7.

## Background

Breast cancer is the most common malignant tumor among females all around the world [1]. Neoadjuvant chemotherapy (NAC) has been widely used in patients with locally advanced breast cancer as an important part of comprehensive breast cancer treatment. Clinical studies had revealed a better prognosis in patients achieving pathologic complete response (pCR) [2]. Thus, it is necessary to find out biomarkers to predict NAC sensitivity. Gamma-glutamyltransferase (GGT) is a membrane-bound enzyme encoded by *GGT1* gene. The previous study had demonstrated it could protect cells from being damaged by radicals and oxidative stress, which is considered to be related to the resistance of antitumor drugs [3]. In our previous study, we had discovered the predictive value of serum GGT in neoadjuvant chemotherapy for breast cancer. Patients with low pre-therapeutic serum GGT levels are more likely to have higher pCR rates, better RFS and DFS, and higher hematologic toxicity [4]. However, the underlying mechanism for the relationship between serum GGT level and NAC sensitivity needs to be further explored.

Single nucleotide polymorphisms (SNPs) are DNA sequence polymorphisms resulting from mutations in a specific nucleotide in the genome of a chromosome. SNPs were reported to be correlated with disease susceptibility as well as drug resistance in vivo [5–9]. The *GGT1* gene, located on human chromosome 22, is the encoding gene of GGT protein. *GGT1* gene. Diergaarde et al. analyzed 26 SNPs and found out that the SNPs rs2017869 and rs8135987 of *GGT1* gene were significantly associated with the incidence and development of pancreatic cancer [3]. Brand et al. reported the correlation between rs8135987 and rs4820599 and the risk of chronic pancreatitis [4]. When it comes to drug toxicity, Khrunin et al. explored the relationship between SNPs rs5751901 and drug toxicity in patients with ovarian cancer and demonstrated that patients with TT genotype of rs5751901 had an increased risk of nephrotoxicity during cisplatin-based chemotherapy [10]. However, there are few studies related to SNPs of *GGT* gene in breast cancer, especially in the neoadjuvant setting.

The serum GGT protein level is affected by both environmental and genetic factors. The correlation between serum GGT protein level and *GGT1* gene SNPs had been identified in different ethnic groups [11, 12]. Melzer et al. found that serum GGT protein level was associated with rs5751901 [11]. The rs4820599 variant in *GGT1* was significantly related to circulating GGT protein level in a large-scale genome-wide association studies (GWAS) meta-analysis in East Asians as well [12].

Based on these premises, we tested a hypothesis that *GGT1* gene SNPs might affect the level of GGT protein in tissues and serum, which would, in turn, affect the efficacy and toxicity of neoadjuvant chemotherapy in breast cancer. We searched for *GGT1* SNPs by using the public database and then did literature research. Finally, three *GGT1* SNPs (rs5751901, rs8135987 and rs2017869), which might have predictive value for disease or prognosis in tumors, were selected in this study. We performed a study to demonstrate whether the SNPs located in *GGT1* gene had an effect on serum GGT protein level and the susceptibility of breast cancer patients undergoing neoadjuvant chemotherapy, in order to find out a genetic marker in predicting NAC sensitivity.

## Method

### Study population

This study consists of 143 newly diagnosed breast cancer patients from December 2013 to January 2018 in Shanghai Jiao Tong university affiliated Renji hospital. All the patients were enrolled in the SHPD001 and SHPD002 clinical trials. The study design and recruitment methods have been described in detail previously [13, 14]. Briefly, all the patients were scheduled to received NAC before surgery. The chemotherapy regimen is a combination of weekly paclitaxel and cisplatin. Human epidermal growth factor receptor-2 (HER-2) positive patients in SHPD001 were recommended to receive concurrent trastuzumab. All HER-2 positive patient in SHPD002 received trastuzumab concomitantly at a weekly basis. For hormone receptor positive patients in SHPD002, endocrine therapy (aromatase inhibitor or gonadotropin releasing hormone agonist) was randomized together with chemotherapy according to their menstrual status.

The pCR (ypT0, defined as the absence of either invasive cancer or cancer in situ in the breast) was used to estimate the efficacy of NAC. HER-2 positive was defined as IHC staining 3 + or FISH (florescent in situ hybridization) showing HER2 gene amplification. Clinical staging was based on the eighth edition of American Joint Committee on Cancer (AJCC) TNM classification. The clinical stage of the patient was determined by CT, MRI and bone scan before treatment. Fine needle or core needle biopsy was performed for clinically significant enlarged lymph nodes. Adverse events were assessed at each visit and recorded according to CTCAE v4.03. Peripheral blood specimens were collected within one week prior to the first cycle of NAC for evaluation of serum GGT and genetic analysis (stored at -80℃). Serum GGT was assayed by the standard method recommended by the International Federation for Clinical Chemistry [15]. The normal range of serum GGT for female was 7–32 U/L at our institution.

### SNP selection and genotyping assays

We selected SNPs by using the public database (NCBI/TargetScan) if they met the following criteria: a) Located in the 3'UTR region or 5'UTR region or intron region of the *GGT1* gene; b) Minimum allele frequency (MAF) > 0.10; c) Reported by other researchers with predicting the value of disease incidence or prognosis.

The *GGT1* gene information of the Han Chinese in Beijing and the Southern Han Chinese using for Hardy–Weinberg equilibrium (HWE) and linkage disequilibrium analysis was obtained using 1000 Genomes Browser.

A total of three SNPs was selected for further studying: rs8135987, rs5751901, rs2017869. Genomic DNA was extracted from the peripheral blood samples using the TIANamp Genomic DNA Kit (Beijing, People’s Republic of China) according to the manufacturer’s protocols (primer sequencing see Supplementary Table 1). The candidate SNPs were genotyped at Shanghai Benegene Biotechnology Co., Ltd (Shanghai, People’s Republic of China), using the MassARRAY system (Sequenom, San Diego, CA, USA).

### Statistical analysis

This study established four genotype models. Assuming A is the major allele and a is the minor allele. The co-dominant model compares AA vs. Aa vs. aa; the dominant model compares AA vs. Aa + aa; the recessive model compares AA + Aa vs. aa; the over-dominant model compares AA + aa vs. Aa [16].

Pearson's chi-square test was used for assessment of Hardy–Weinberg equilibrium (HWE) [17] and the frequency differences in the genotype and haplotype distribution between groups (different pCR outcomes and serum GGT levels). Multivariable logistic regression analyses were used to analyze the associations between different SNP genotypes, haplotypes and pCR outcomes, serum GGT level as well as adverse events. Results were shown as p-value, odds ratio (OR), and 95% confidence interval (95%CI). A two-sided p value < 0.05 was considered statistically significant. Stata SE 14.1 (Stata Corp LP, USA) was used in statistical analysis. Haploview 4.1 and PHASE 2.1 was used for haplotype analysis.

## Results

### Genotype distributions

**Fig. 1:**
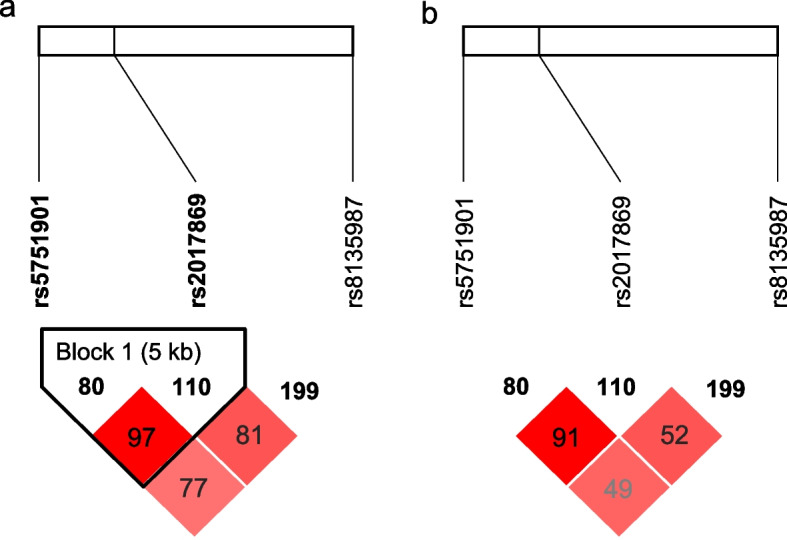
D and r^2^ value of paired *GGT1* gene SNP loci. **a** D value of paired SNP loci. **b** r^2^ value of paired SNP loci

No deviations from HWE were detected (p > 0.05) (Table 1). SNPs rs5751901 was in linkage disequilibrium with rs2017869 (D' = 0.97, r2 = 0.91) (Fig. 1), further haplotype analysis were carried out with these two loci. A total of 143 patients were included in this study.

**Table 1:** Genotype distribution and HWE

Gene	SNP	SNP location	Major allele	Minor allele	Genotype Distribution ^A^ N (%)	MAF	HWE
***GGT1* gene**	rs8135987	chr22: 25,012,854	T	C	69(48.3)/58(40.5)/16(11.2)	0.303	0.1546
	rs5751901	chr22: 24,992,266	T	C	57(39.9)/62(43.3)/24(16.8)	0.344	0.1029
	rs2017869	chr22: 24,997,309	G	C	59(41.3)/61(42.6)/23(16.1)	0.353	0.0687

*HWE* Hardy–Weinberg equilibrium, *MAF* minor allele frequency (Southern Han Chinese and Han Chinese in Beijing)

^A^ The order of genotype is wild homozygote/heterozygote/mutant homozygote

### Clinic-pathological characteristics and the relationship between three SNPs and the efficacy of NAC

The average age of all patients was 51.5 years (range, 26–70 years). A total of 50 patients achieved pCR in NAC with a pCR rate of 34.97% (Table 2). For the SNP rs8135987, patients with CC + TC genotype had significantly lower pCR rate compared with the TT genotype in the dominant model (*p* = 0.039). Results from the multivariable logistic regression analysis revealed that the odds of obtaining pCR for TC genotype were 0.30 times the odds of obtaining pCR for TT genotype in co-dominant model (95% CI 0.09–0.90, *p* = 0.033) and 0.30 times the odds of obtaining pCR for TT + CC genotype in over-dominant model (95% CI 0.10–0.88, *p* = 0.029). In other words, TC genotype was less likely to obtain pCR in both co-dominant model and over dominant model (Table 3).

**Table 2:** Clinic-pathological characteristics of the study population

Patient characteristics	N(%)
Age at first diagnosis (years)	
≤ 50	61 (42.66)
> 50	82 (57.34)
Menses	
Premenopausal	59(41.26)
Postmenopausal	84(58.74)
Body Mass Index (BMI)	
≤ 23	69(48.25)
> 23	74(51.75)
Estrogen Receptor (ER) (%)	
< 10	50(34.97)
≥ 10	93(65.03)
Progesterone Receptor (PR) (%)	
< 10	42(29.37)
≥ 10	101(70.63)
Ki-67 (%)	
< 30	35(24.48)
≥ 30 & < 60	65(45.45)
≥ 60	43(30.07)
HER-2 status^A^	
Negative	84(58.74)
Positive	59(41.26)
Clinical Tumor stage^B^	
cT1-cT2	72(50.35)
cT3-cT4	71(49.65)
Clinical Nodal status^B^	
cN0	19(13.29)
cN1-3	124(86.71)
Efficacy	
Non-pCR	93(65.03)
pCR	50(34.97)

^A^HER-2 positive was defined as IHC staining 3 + or FISH (florescent in situ hybridization) showing HER2 gene amplification

^B^The clinical staging was based on the eighth edition of American Joint Committee on Cancer (AJCC) TNM classification. The clinical stage of the patient was determined by CT, MRI and bone scan before treatment. Fine needle or core needle biopsy was performed for clinically significant enlarged lymph nodes

**Table 3:** Association between selected SNPs and pCR

	**pCR group**	**Non-pCR group**	**χ**^**2**^	**Pearson p**^**A**^	**Logit p**^**B**^	**OR(95%CI) **^**C**^
	N(%)	N(%)				
**Genotype Distribution**						
** rs8135987 (T > C)**						
** TT**	30(60.0)	39(41.9)	-	-	-	-
** TC**	16(32.0)	42(45.2)	3.450	0.063	**0.033**	0.30(0.09–0.90)
** CC**	4(8.0)	12(12.9)	1.848	0.258	0.976	1.03(0.14–7.14)
** TT**	30(60.0)	39(41.9)	-	-	-	-
** TC + CC**	20(40.0)	54(58.1)	4.250	**0.039**	0.060	0.37(0.13–1.04)
** TT + TC**	46(92.0)	81(87.1)	-	-	-	-
** CC**	4(8.00)	12(12.9)	0.787	0.422	0.591	1.64(0.26–10.10)
** TT + CC**	34(68.0)	51(54.8)		-	-	-
** TC**	16(32.0)	42(45.1)	2.336	0.126	**0.029**	0.30(0.10–0.88)
** Allele**						
** C**	24(24.0)	66(35.5)	-	-	-	-
** T**	76(76.0)	120(64.5)	3.977	**0.046**	-	-
** rs5751901 (T > C)**						
** TT**	21(42.0)	36(38.7)	-	-	-	-
** TC**	20(40.0)	42(45.2)	0.276	0.599	0.475	0.68 (0.23–1.95)
** CC**	9(18.0)	15(16.1)	0.003	0.955	0.629	1.42 (0.33–5.96)
** TT**	21(42.0)	36(38.7)	-	-	-	-
** TC + CC**	29(58.0)	57(61.2)	0.147	0.702	0.705	0.83(0.31–2.18)
** TT + TC**	41(82.0)	78(83.9)	-	-	-	-
** CC**	9(18.0)	15(26.1)	0.081	0.817	0.436	1.67(0.44–6.31)
** TT + CC**	30(60.0)	51(54.8)	-	-	-	-
** TC**	20(40.0)	42(45.2)	0.352	0.553	0.352	0.62(0.23–1.68)
** Allele**						
** C**	38(38.0)	72(38.7)	-	-	-	-
** T**	62(62.0)	114(61.3)	0.138	0.906	-	-
** rs2017869 (G > C)**						
** GG**	21(42.0)	38(40.8)	-	-	-	-
** GC**	21(42.0)	40(43.0)	0.018	0.893	0.800	0.87(0.31–2.49)
** CC**	8(16.0)	15(16.1)	0.005	1.000	0.616	1.45(0.34–6.23)
** GG**	21(42.0)	38(40.9)	-	-	-	-
** GC + CC**	29(58.0)	55(59.1)	0.017	0.895	0.998	0.99(0.37–2.61)
** GG + GC**	42(84)	78(83.9)	-	-	-	-
** CC**	8(16)	15(16.1)	0.000	1.000	0.537	1.50(0.38–6.11)
** GG + CC**	29(58.0)	53(57.0)	-	-	-	-
** GC**	21(42.0)	40(43.0)	0.014	0.907	0.662	0.80(0.29–2.16)
** Allele**						
** G**	63(63.0)	116(62.4)	-	-	-	-
** C**	37(37.0)	70(37.6)	0.011	0.916	-	-
** Haplotype (rs5751901—rs2017869)**						
** TG**	42(58.3)	78(58.2)	0.000	0.984	-	-
** CC**	28(38.9)	55(41.0)	0.132	0.717	-	-
** CG**	2(2.8)	1(0.8)	0.004	0.952	-	-

^A^Pearson χ^2^ test

^B^*P* values were analyzed with adjustment for age, BMI, ER, PR, HER-2, Ki-67, clinical T stage, and clinical N stage

^C^OR and 95%CI were analyzed by multivariable logistic regression

For SNP rs5751901 and rs2017869, no significant evidence supported a correlation between SNP genotypes, haplotypes and pCR outcomes (Table 3).

### Relationship between three SNPs and adverse events of NAC

A total of 106 patients with available data of adverse events were included in analysis. For SNP rs2017869, multivariable analysis showed that the odds of neutropenia for CC genotype was 0.39 times the odds of neutropenia for TT + TC genotype (95% CI 0.08–0.84, *p* = 0.025) in recessive model. The the odds of leukopenia for CC genotype was 0.24 times the odds of leukopenia for TT + TC genotype (95% CI 0.08–0.78, *p* = 0.017) in recessive model. For SNP rs5751901, the CC genotype decreased the risk of ouccuring neutropenia (adjusted OR = 0.29, 95% CI 0.09–0.96, *p* = 0.036) and leukopenia (adjusted OR = 0.27, 95% CI 0.09–0.84, *p* = 0.024) compared with TT + TC genotype in the recessive model. For SNP rs8135987, it was pronounced correlated with grade 2 or greater peripheral neuropathy, the risk of which was significantly lower in TC genotype compared with the TT + CC genotype in the over-dominant model (adjusted OR = 0.39, 95% CI 0.15–0.96, *p* = 0.042). In addition, the CC genotype in the recessive model was an independent protective factor for leukopenia (adjusted OR = 0.16, 95% CI 0.05–0.68, *p* = 0.014), while it was an independent risk factor for elevated AST (adjusted OR = 4.5, 95% CI 1.11–18.27, *p* = 0.035). The detailed adverse events spectrum is shown in Table 4. In haplotype analysis for rs5751901 and rs2017869, the occurance of grade 2 or greater neutropenia for TG haplotype was increased by 3.05 times (adjusted OR = 4.05, 95% CI 1.27–12.92, *p* = 0.018) and the occurance of grade 2 or greater leukopenia for TG haplotype was increased by 3.26 times (adjusted OR = 4.26, 95% CI 1.36–13.30, *p* = 0.013) (Table 5).

**Table 4:** Association between SNPs and patients’ adverse events during NAC

Toxic reactions	SNP	Genotypes	Toxicity grade		OR(95%CI)	p^A^
**Neutropenia**			Grade < 2	Grade ≥ 2		
	**rs8135987 (T > C)**	**TT**	9(42.9)	38(44.7)		
		**TC**	7(33.3)	39(45.9)	1.66(0.52–5.24)	0.391
		**CC**	5(23.8)	8(9.4)	0.47(0.11–2.04)	0.314
		**TT vs. TC + CC**			1.15(0.42–3.18)	0.790
		**TT + TC vs. CC**			0.38(0.09–1.54)	0.175
		**TT + CC vs. TC**			1.96(0.66–5.83)	0.227
	**rs5751901 (T > C)**	**TT**	6(28.6)	36(42.4)		
		**TC**	8(38.1)	38(44.7)	0.70(0.25–2.96)	0.824
		**CC**	7(33.3)	11(12.9)	0.26(0.07–1.01)	0.052
		**TT vs. TC + CC**			0.56(0.19–1.67)	0.300
		**TT + TC vs. CC**			0.29(0.09–0.96)	**0.036**
		**TT + CC vs. TC**			1.43(0.49–4.12)	0.504
	**rs2017869 (G > C)**	**GG**	7(33.3)	37(43.5)		
		**GC**	7(33.3)	38(44.7)	1.23(0.36–4.20)	0.740
		**CC**	7(33.3)	10(11.8)	0.28(0.08–1.04)	0.059
		**GG vs. GC + CC**			0.70(0.24–2.03)	0.514
		**GG + GC vs. CC**			0.39(0.08–0.84)	**0.025**
		**GG + CC vs. GC**			1.90(0.63–5.72)	0.251
**Leukopenia**			Grade < 2	Grade ≥ 2		
	**rs8135987 (T > C)**	**TT**	8(33.3)	39(47.6)		
		**TC**	9(37.5)	37(45.1)	0.85(0.36–4.20)	0.765
		**CC**	7(29.2)	6(7.3)	0.16(0.05–0.68)	**0.014**
		**TT vs. TC + CC**			0.54(0.19–1.47)	0.229
		**TT + TC vs. CC**			0.17(0.04–0.67)	**0.012**
		**TT + CC vs. TC**			1.30(0.48–3.52)	0.602
	**rs5751901 (T > C)**	**TT**	8(33.3)	34(41.5)		
		**TC**	8(33.3)	38(46.3)	0.99(0.31–3.13)	0.998
		**CC**	8(33.3)	10(12.2)	0.27(0.07–0.96)	**0.045**
		**TT vs. TC + CC**			0.63(0.23–1.71)	0.363
		**TT + TC vs. CC**			0.27(0.09–0.84)	**0.024**
		**TT + CC vs. TC**			1.59(0.58–4.34)	0.370
	**rs2017869 (G > C)**	**GG**	9(37.5)	35(42.7)		
		**GC**	7(29.2)	38(46.3)	1.29 (0.40–4.13)	0.663
		**CC**	8(33.3)	9(11.0)	0.27 (0.08–0.97)	**0.045**
		**GG vs. GC + CC**			0.71(0.27–1.95)	0.523
		**GG + GC vs. CC**			0.24(0.08–0.78)	**0.017**
		**GG + CC vs. GC**			1.97(0.69–5.64)	0.204
**Peripheral neuropathy**			Grade < 2	Grade ≥ 2		
	**rs8135987 (T > C)**	**TT**	26(39.4)	21(52.5)		
		**TC**	34(51.5)	12(30.0)	0.43(0.17–1.09)	0.078
		**CC**	6(9.1)	7(17.5)	1.70(0.41–7.07)	0.459
		**TT vs. TC + CC**			0.60(0.26–1.40)	0.236
		**TT + TC vs. CC**			2.42(0.61–9.59)	0.206
		**TT + CC vs. TC**			0.39(0.15–0.96)	**0.042**
	**rs5751901 (T > C)**	**TT**	27(40.9)	15(37.5)		
		**TC**	30(45.5)	16(40.0)	1.95(0.59–6.45)	0.270
		**CC**	9(13.6)	9(22.5)	0.96(0.37–2.47)	0.928
		**TT vs. TC + CC**			1.19 (0.50–2.85)	0.688
		**TT + TC vs. CC**			2.00(0.67–5.96)	0.213
		**TT + CC vs. TC**			0.77(0.32–1.83)	0.561
	**rs2017869 (G > C)**	**GG**	28(42.4)	16(40.0)		
		**GC**	29(43.9)	16(40.0)	0.96(0.37–2.49)	0.939
		**CC**	9(13.6)	8(20.0)	0.69(0.50–5.65)	0.393
		**GG vs. GC + CC**			1.15(0.48–2.73)	0.757
		**GG + GC vs. CC**			1.72(0.56–5.25)	0.340
		**GG + CC vs. GC**			0.83(0.34–1.98)	0.668
**AST increased**			Grade < 1	Grade ≥ 1		
	**rs8135987 (T > C)**	**TT**	29(51.8)	18(36.0)		
		**TC**	22(29.3)	24(48.0)	1.50(0.60–3.66)	0.386
		**CC**	5(8.9)	8(16.0)	5.40(1.24–23.4)	**0.024**
		**TT vs. TC + CC**			1.94(0.84–4.50)	0.122
		**TT + TC vs. CC**			4.50(1.11–18.27)	**0.035**
		**TT + CC vs. TC**			1.09(0.46–2.53)	0.844
	**rs5751901 (T > C)**	**TT**	25(44.6)	17(34.0)		
		**TC**	22(39.3)	24(48.0)	1.67(0.50–5.47)	0.401
		**CC**	9(16.1)	9(18.0)	1.28(0.51–3.20)	0.591
		**TT vs. TC + CC**			1.39(0.59–3.22)	0.448
		**TT + TC vs. CC**			1.47(0.49–4.41)	0.493
		**TT + CC vs. TC**			1.10(0.48–2.55)	0.818
	**rs2017869 (G > C)**	**GG**	26(49.1)	18(36.0)		
		**GC**	22(39.3)	23(46.0)	1.13(0.45–2.86)	0.784
		**CC**	8(14.3)	9(18.0)	1.96(0.58–6.60)	0.279
		**GG vs. GC + CC**			1.34(.057–3.10)	0.500
		**GG + GC vs. CC**			1.84(4.58–5.77)	0.293
		**GG + CC vs. GC**			0.95(0.40–2.23)	0.904

*AST* Aspartate transaminase

^A^*P* values were analyzed with adjustment for age, BMI, menses, ER, PR, if accepted Herceptin or not

**Table 5:** Occurrence of adverse events during NAC according to haplotypes

Haplotype	N(%)		χ^2^	Pearson p^A^	Logit p^B^	OR(95%CI) ^C^
TG	89 (61.8)					
CC	61 (36.8)					
CG	3 (1.4)					
Neutropenia	**Grade < 2**	**Grade ≥ 2**				
TG	14 (15.7)	75(84.3)	5.818	**0.016**	**0.018**	4.05(1.27–12.92)
CC	14(22.9)	47(77.1)	0.892	0.345	0.413	0.65(0.23–1.83)
CG	1(33.3)	2(66.7)	0.355	0.551	0.393	0.33(0.03–4.23)
Leukopenia	**Grade < 2**	**Grade ≥ 2**				
TG	16(18.0)	73(82.0)	6.892	**0.009**	**0.013**	4.26(1.36–13.30)
CC	15(24.6)	46(75.4)	0.312	0.577	0.404	0.65(0.24–1.77)
CG	1(33.3)	2(66.7)	0.202	0.541	0.605	0.51(0.34–6.56)
Peripheral neuropathy	**Grade < 2**	**Grade ≥ 2**				
TG	58(65.2)	31(34.8)	1.992	0.158	0.142	0.43(0.14–1.32)
CC	37(60.7)	24(39.3)	0.158	0.691	0.512	1.34(0.56–3.21)
CG	1(33.3)	2(66.7)	1.100	0.294	0.371	3.18(0.25–40.30)
AST increased	**Grade < 1**	**Grade ≥ 1**				
TG	48 (53.9)	41(46.1)	0.271	0.603	0.411	0.63(0.21–1.89)
CC	30(49.2)	31(50.8)	0.768	0.381	0.596	1.25(0.55–2.88)
CG	2(66.7)	1(33.3)	0.237	0.626	0.602	0.51(0.39–6.59)

^A^Pearson χ^2^ test

^B^*P* values were analyzed with adjustment for age, BMI, menses, ER, PR, if accepted Herceptin or not

^C^OR and 95%CI were analyzed by multivariable logistic regression

### GGT SNPs and serum GGT level

Patients were divided into a high-level group (≥ 29 U/L) and a low-level group (< 29 U/L) according to the serum GGT level according to our previous study [5]. For SNP rs8135987, the TC genotype of both the dominant model (adjusted OR = 3.11, 95% CI 1.07–9.02, *p* = 0.036) and the co-dominant model (adjusted OR = 2.20, 95% CI 1.25–12.62, *p* = 0.019) were significantly associated with elevated serum GGT level in multivariable analysis. For SNPs rs2017869, the CC genotype of the recessive model was significantly related with higher serum GGT level (adjusted OR = 3.09, 95% CI 1.02–9.36, *p* = 0.046) in multivariable analysis. For SNPs rs5751901, the distribution of the CC genotype of co-dominant model (*p* = 0.034) and recessive models (*p* = 0.028) were both significantly difference among GGT levels, while the difference was not statistically significant in multivariable analysis (Table 6).

**Table 6:** Association between SNPs and pre-therapeutic serum GGT level

Genotypes	Serum GGT level N (%)		p^A^	p^B^	OR(95%CI) ^C^
	Low level < 29 U/L	High level ≥ 29U/L			
**rs8135987 (T > C)**					
** TT**	60(52.6)	9(31.0)			
** TC**	43(37.7)	15(51.7)	0.073	**0.019**	2.20(1.25–12.62)
** CC**	11(9.7)	5(17.3)	0.127	0.351	3.97(0.41–11.59)
** TT vs. TC + CC**			0.060	**0.036**	3.11(1.07–9.02)
** TT + TC vs. CC**			0.319	0.791	1.22(0.27–5.45)
** TT + CC vs. TC**			0.170	0.050	2.76(0.99–7.61)
**rs5751901 (T > C)**					
** TT**	49(43.0)	8(27.6)			
** TC**	50(43.9)	12(41.4)	0.472	0.497	1.48(0.47–4.64)
** CC**	15(13.1)	9(31.0)	**0.034**	0.068	3.32(0.91–12.13)
** TT vs. TC + CC**			0.144	0.262	1.79(0.65–4.98)
** TT + TC vs. CC**			**0.028**	0.067	2.78(0.93–8.29)
** TT + CC vs. TC**			0.810	0.714	0.84(0.32–2.17)
**rs2017869 (G > C)**					
** GG**	50(43.8)	9(31.0)			
** GC**	50(43.8)	11(38.0)	0.808	0.825	3.32(0.93–11.91)
** CC**	14(12.4)	9(31.0)	**0.035**	0.065	1.14(0.36–3.54)
** GG vs. GC + CC**			0.291	0.442	1.47(0.54–3.96)
** GG + GC vs. CC**			**0.022**	**0.046**	3.09(1.02–9.36)
** GG + CC vs. GC**			0.564	0.411	0.67(0.25–1.76)
**Haplotype**					
** TG**			0.059	0.157	0.44(0.14–1.37)
** CC**			0.361	0.477	1.44(0.53–3.95)
** CG**			0.570	0.677	1.74(0.13–23.40)

^A^Pearson χ^2^ test

^B^*P* values were analyzed with adjustment for age, BMI, ER, PR, HER-2, Ki-67, clinical T stage, and clinical N stage

^C^OR and 95%CI were analyzed by logistic logistic regression

## Discussion

To the best of our knowledge, this study is the first one to explore the relationship between *GGT1* gene SNPs and NAC efficacy and adverse events, as well as serum GGT levels in breast cancer. Here we found out that *GGT1* gene SNPs have potential value in predicting the efficacy and tolerability of neoadjuvant chemotherapy. In our findings, we revealed for the first time the relationship between SNP rs8135987 (T > C) and NAC efficacy, adverse events, and serum GGT level. The TC genotype of SNP rs8135987 showed negative relation to pCR in both the over-dominant model and co-dominant model. Furthermore, we found that the TC genotype of SNP rs8135987 was an independent protective factor for the occurrence of peripheral neuropathy. Thus, the TC genotype of rs8135987 may be a novel biomarker of the resistance to NAC, which may lead to reduced treatment sensitivity as well as the incidence of adverse events.

Based on previous studies, SNPs in and near *GGT1* gene could influence the expression level and activity of serum GGT [18, 19]. The observed genetic covariance in a twin study indicated that plasma GGT levels may caused by genetics [20]. The serum GGT is extremely important in mediating the intracellular glutathione (GSH) levels. The most critical biological function of GSH is anti-oxidation and neutralizing free radicals [21]. Thus, serum GGT plays a vital role in protecting the cell against oxidative stress and further resisting the toxicity of the promoting agents, which means it may enhance the resistance to pro-oxidant cancer therapy [22]. Several previous studies had confirmed that GGT can influence the sensitivity of tumor cells to drugs [3, 23]. Our previous study also illuminated the predictive value of serum GGT level in NAC [5]. In this study, we found that the TC genotype of rs8135987 was associated with elevated GGT level. It may revealed the underlying mechanisms of the insensitivity of TC genotype of SNP rs8135987 to NAC in breast cancer. In addition, the recessive models of rs2017869 and rs5751901 were both significantly associated with pre-treatment serum GGT levels, consisting with the results of other research [10], which found that each minor allele of rs5751901 was associated with a 0.21 standard deviation increase in GGT1 protein level. Moreover, Sciskalska et al. also found that SNP rs5751901 may cause changes in GGT activity. The TC genotype for SNP rs5751901 had an increased blood GGT activity compared to with CC genotypes in smokers [24].

Our study was the first to find out the relationship between *GGT1* gene SNPs and the neurotoxicity and hematotoxicity of chemotherapy. In this study, we found that TC genotype of SNP rs8135987 was an independent protective factor for the occurrence of peripheral neuropathy. What’s more, significant negative correlation exists between the recessive models of rs2017869, rs5751901 and neutropenia as well as leukopenia, suggesting that SNPs rs2017869 and rs5751901 have certain predictive value for patient’s tolerability of NAC. Haplotype analysis also confirmed this result, the TG haplotype of rs5751901 and rs2017869 had an increased occurrence of neutropenia and leukopenia. Practically, the tolerability to chemotherapy may be related to the metabolism of the drugs in vivo. Khrunin et al. found that the TT genotype of rs5751901 was significantly associated with cisplatin nephrotoxicity in patients with ovarian cancer. The frequency of TT genotype was approximately 50% in patients with renal failure, while it was only 31% in those with normal renal function [9]. However, all patients in our study received small-dose weekly cisplatin combined with paclitaxel, none of them had severe renal impairment, and that might be why there was no significant correlation between the *GGT1* gene SNPs and elevated creatinine (Supplementary Table 2).

The selected SNP loci fall within the non-coding regions of GGT1 gene. As we know, introns’ sequences account for nearly 24% of the entire human genome [25]. The human genome contains millions of SNPs and many of them are intronic and have unknown functional significance. Previous study showed that intron-located SNPs affect splicing, alternative splicing and splicing efficiency and confer risk for the development of different multifactorial human diseases [26–29]. A GWAS study suggested that SNPs located in the intron may as well alter the protein levels by the linkage disequilibrium with adjacent alleles or regulating gene expression in ways of affecting mRNA splicing. SNP rs5751901 was found to be in linkage disequilibrium with rs6519519 (r^2^ = 0.71), which was related to the transcript abundance of *GGT1* gene [11]. SNP rs5751901 was also in linkage disequilibrium with rs4820599, which was located in the *GGT1* gene transcript region as a potential transcriptional binding site [18]. SNP rs8135987 is located in an intron of *GGT1* gene and within 2 kb of exon 9, which is found to be crucial for substrate binding and catalysis [30]. These might be the potential biological mechanisms by which these non-coding SNPs affected serum GGT protein level and thus affect pCR outcome and adverse events. This hypothesis might be further studied and validated in the future.

A limitation of our study is the relatively small sample size, which might undermine the statistical power. However, since the object of the study was the patients undergoing prospective clinical studies, who have complete and highly reliable clinical and pathological information, our results can prompt an inherent law for predicting chemotherapeutic sensitivity. Nevertheless, it is necessary to expand the number of the samples or to conduct multi-center prospective studies in the future, so as to better verify the predictive value of *GGT1* gene varieties in NAC of breast cancer.

In summary, there is convincing evidence that *GGT1* gene SNPs were potential markers in predicting the therapeutic effect and adverse events of NAC. Moreover, the correlation between *GGT1* gene SNPs and serum GGT protein level was verified. This study represents a step forward toward a better understanding of the effect of *GGT1* gene in breast cancer, providing a theoretical and clinical basis for individualized treatment.

## Conclusions

The *GGT1* gene SNPs might be a novel biomarker of the sensitivity of NAC in breast cancer patients, providing theoretical basis for further precision therapy.

### Supplementary Information

**Additional file 1:** **Supplementary Table 1.** Primer of GGT SNPs. **Supplementary Table 2.** Association between SNPs and elevated creatinine during NAC

## Data Availability

The data that support the findings of this study are available from the corresponding author, Jingsong Lu, upon reasonable request.

## Abbreviations

GGT: Gamma-glutamyl transpeptidase
*G**GT1*: Gamma-glutamyltransferase 1
SNP: Single nucleotide polymorphisms
NAC: Neoadjuvant chemotherapy
pCR: Pathologic complete response
GSH: Glutathione
BMI: Body mass index
ER: Estrogen receptor
PR: Progesterone receptor
