# Supplementary material for: Genetic polymorphisms of GGT1 gene (rs8135987, rs5751901 and rs2017869) are associated with neoadjuvant chemotherapy efficacy and toxicities in breast cancer patients
Source: BMC Med Genomics. 2023 Oct 27;16:267. doi: 10.1186/s12920-023-01685-7 (PMC10612355; doi:10.1186/s12920-023-01685-7)
Supplement: Supplementary file 1 — Additional file 1: Supplementary Table 1. Primer of GGT SNPs. Supplementary Table 2. Association between SNPs and elevated creatinine during NAC [file 12920_2023_1685_MOESM1_ESM.docx]

Supplementary Table1 Primer of GGT SNPs

| rs Number | Primer sequence |
| --- | --- |
| rs5751901 |  |
| Primer 1 | ACGTTGGATGTGGAGTCCTCAAAGGGAGTG |
| Primer 2 | ACGTTGGATGGCAACAGAATAGTTCTGGAG（R） |
| rs8135987 |  |
| Primer 1 | ACGTTGGATGCTGTGCCCGATCAATCTTTC |
| Primer 2 | ACGTTGGATGTCCCGCATTCTCTTACCCTG（F） |
| rs2017869 |  |
| Primer 1 | ACGTTGGATGAGGTCCAAGAAGCTGACAAC |
| Primer2 | ACGTTGGATGTCACCATGGCCCACTCAGG（R） |

Supplementary Table2 Association between SNPs and elevated creatinine during NAC

| **Toxic reactions** | **SNP** | **Genotypes** | **Toxicity grade** | | **OR（95%CI）** | **p^A^** |
| --- | --- | --- | --- | --- | --- | --- |
| **SCr increased** |  |  | Grade <1 | Grade ≥1 |  |  |
|  | **rs8135987 (T>C)** | **TT** | 23(37.7) | 24(53.3) |  |  |
|  |  | **TC** | 29(47.5) | 17(37.8) | 0.67(0.28-1.59) | 0.369 |
|  |  | **CC** | 9(14.8) | 4(8.9) | 0.40(0.10-1.59) | 0.193 |
|  |  | **TT vs. TC+CC** |  |  | 0.60(0.27-1.34) | 0.211 |
|  |  | **TT+TC vs. CC** |  |  | 0.47(0.12-1.70) | 0.268 |
|  |  | **TT+CC vs. TC** |  |  | 0.79(0.34-1.81) | 0.576 |
|  | **rs5751901 (T>C)** | **TT** | 21(34.4) | 21(46.7) |  |  |
|  |  | **TC** | 29(47.6) | 17(37.8) | 0.64(0.26-1.56) | 0.324 |
|  |  | **CC** | 11(18.0) | 7(15.5) | 0.63(0.20-2.02) | 0.440 |
|  |  | **TT vs. TC+CC** |  |  | 0.64(0.28-1.45) | 0.281 |
|  |  | **TT+TC vs. CC** |  |  | 0.79(0.27-2.31) | 0.667 |
|  |  | **TT+CC vs. TC** |  |  | 0.73(0.32-1.67) | 0.455 |
|  | **rs2017869 (G>C)** | **GG** | 23(37.7) | 21(46.7) |  |  |
|  |  | **GC** | 28(45.9) | 17(37.8) | 0.75(0.30-1.85) | 0.534 |
|  |  | **CC** | 10(16.4) | 7(15.5) | 0.75(0.23-2.41) | 0.634 |
|  |  | **GG vs. GC+CC** |  |  | 0.75(0.33-1.71) | 0.496 |
|  |  | **GG+GC vs. CC** |  |  | 0.86(0.28-2.56) | 0.785 |
|  |  | **GG+CC vs. GC** |  |  | 0.81(0.35-1.89) | 0.629 |

^A^ Pearson χ² test

^B^ P values were analyzed with adjustment for age, BMI, ER, PR, HER-2, Ki-67, clinical T stage, and clinical N stage

^C^ OR and 95%CI were analyzed by logistic logistic regression.
